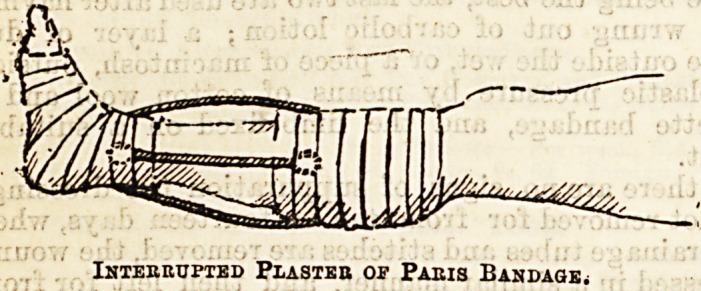# Treatment of Compound Fractures

**Published:** 1893-08-19

**Authors:** 


### THE HOSPITAL CLINIC.

[*The Editor will be glad to receive offers of co-operation and contributions from members of the profession. All letters should be addressed to* THE EDITOR, THE LODGE, PORCHESTER SQUARE, LONDON, W.]

### THE LONDON HOSPITAL.

### TREATMENT OF COMPOUND FRACTURES.

Success in the treatment of compound fracture depends chiefly on the extent to which the wound can be kept aseptic, and on the completeness with which rest can be secured for the injured part.

When a compound fracture is first seen, care is taken to exclude the wound as soon as possible from the air, and to put the parts at rest. As soon as the fact of a compound fracture being present is made out, further interference, except to stop bleeding, is avoided. The injured limb is placed on a temporary splint, usually some form of stiff pillow, or between sand bags, and the wound covered with lint saturated with carbolic water (1-20). A thorough examination of every compound fracture is necessary, one examination only should be made—made antiseptically and thoroughly. Bleeding, of course, is stopped at once if deep and severe, the elastic tourniquet is used as the quickest and simplest; but as it necessarily stops the circulation completely in the limb, and so lowers the vitality of the injured part, it is used as little as possible; pressure forceps being used instead, when the bleeding point can be easily seen and got at. After the "first aid" the treatment depends on the nature and situation of the fracture, and to a great extent on the previous habits of the patient; not only is the wound far more likely to suppurate in those who have been in the habit of unduly indulging in alcohol, but the well known tendency of such patients to become delirious after an injury makes it necessary to take increased pains to secure immobility of the affected limb.

The simplest cases are those where there is only a small skin wound, free from dirt, and the bone underneath broken across, but not splintered. The open method is generally used in these cases. After the limb is put in splints in the usual way, the wound having been thoroughly washed out with 1-1000th perchloride of mercury solution, it is thickly powdered with iodoform or creolin powder, and left exposed to the air. If pus forms it can be seen at once; should serous discharge leak through, more iodoform or creolin is powdered on, but the wound is not again opened. This treatment gives excellent results in suitable cases, the wound being constantly under observation, though sealed by an antiseptic from the air; any sign of suppuration can be detected at once without having to disturb the limb by the removal of dressings. In more severe cases either wiring or pegging the broken ends of the bones together is done, with good results, provided the main artery of the limb is uninjured. An anæsthetic having been administered, the skin being cleansed as thoroughly as possible, a free incision is made exposing the seat of fracture, if the original skin wound is not large enough. Clots and dirt are then removed with the greatest care, as on the thoroughness with which this is done depends the success of the operation.

Any bone from which the periosteum has been stripped is removed with bone forceps, as well as any loose splinters. The wound is again washed out with perchloride of mercury solution (1-1000) till all trace of clot or dirt is removed. The two ends of the bone being now brought together in their natural position, a hole is drilled through the two—either a piece of strong silver wire passed through the hole, brought up round one side of the bone, the two ends twisted up till the wire is tight, and the ends left projecting outside the skin, or an ivory peg is driven into the drill-hole, and cut off flush with the surface of the bone. The wound being again carefully washed out, counter-openings are made at the most dependent part of the wound cavity for drainage, perforated rubber drainage tubes being used.

The wound is closed through the greater part of its extent with silkworm gut sutures, all bleeding vessels having been previously stopped by torsion or ligature. One of the antiseptic gauze dressings is now applied, Tillman's, salalembroth, or bicyanide of mercury gauze being the best, the last two are used after having been wrung out of carbolic lotion; a layer of dry gauze outside the wet, or a piece of macintosh, outside all elastic pressure by means of cotton wool and a domette bandage, and the limb fixed on a suitable splint.

If there are no signs of suppuration the dressings are not removed for from ten to fourteen days, when the drainage tubes and stitches are removed, the wound redressed in a similar manner, and then left for from six to eight weeks, with occasional redressing at intervals of about ten to fourteen days if there is any discomfort.

At the end of that time it is necessary to remove the

Limb fixed in an interrupted splint suspended from cradle—resting on end of mattress above—block or sandbag below—antiseptic fluid from pail hung on top of bed leads to a glass 3-way tube—into the wound at upper part—out at lower opening and drops into pail under bed—3 way tube being omitted if only one tube is necessary.

wire, the twisted ends of which are still projecting from the skin. The wire is untwisted, one end cut off as close as possible to the bone with wire nippers, a pull on the other end removes the wire without much pain or difficulty.

Occasionally the wire after being twisted is hammered down flat and left permanently in the wound, but on the whole it has been found the best to remove the wire, after good union of the bones has taken place. If an ivory peg has been used removal is not necessary, but on the other hand, the bones are at first not as firmly fixed as when wire is used, and in a considerable number of cases the shape of the fracture is such as to render successful pegging difficult or impossible. The methods of securing immobility of the limb vary of course according to the situation of the fracture; in most cases removable wooden splints of some form or other are used, but where, either from delirium or other couses, a patient is very restless, plaster of paris in some form is used, very complete immobility being thus obtained, but with the disadvantage that the surface of the limb is hidden from sight.

Plaster of paris is used for this purpose in two ways, either applied as a "croft," consisting of a piece of Bavarian felt cut roughly to envelope half the limb, soaked in freshly mixed plaster of paris and water, and applied between two pieces of lint rather larger than the felt; a second piece being applied to the other side of the limb in a similar manner, so that the edges of the two pieces overlap. This dries quickly, is taken off, the edges trimmed, and a "window" cut out over the site of the wound so that dressing can be applied, and the wound watched, without disturbing the splints. Coarse muslin bandages rubbed with plaster of paris

Interrupted Plaster of Paris Bandage.

are also used, wetted, and applied over a domette bandage above and below the fracture. Three or four strips of sheet iron, or thick iron wire, bent in the middle so as not to be quite close to the skin, pass lengthways down the limb from above to below the fracture; their ends, which are either flattened out or looped to give a firm hold for the plaster being included in the folds of the bandages, and the whole limb slung from a cradle.

Sometimes poroplastic felt is used and makes an excellent splint, which when softened by heat, can be moulded to the exact shape of the limb.

For compound fractures about the hand or foot the continuous bath is often used. The hand or foot, as the case may be, being kept either constantly immersed in a bath of boracic acid lotion or solution of perchloride of mercury (1-10,000); or else kept in the bath from eight to twelve hours daily, the remainder of the time either boracic fomentations being applied to remove sloughs, or the part dressed with some antiseptic gauze—salalembroth, perchloride or bicyanide of mercury being most often used. When suppuration has lessened, and the wound is covered with healthy granulations, ordinary dry antiseptic dressing, such as wood wool or gamgee, with iodoform, is used, being alternated with or replaced by astringent and stimulating lotions, such as nitrate of silver or sulphate of zinc, if the granulations show a tendency to become too soft or flabby.

The principle of this form of treatment, namely, constantly washing away pus or septic matter, is applied in a slightly different manner to compound fractures in the larger bones, where the size of the limb renders constant immersion a practical impossibility.

The limb being fixed in as good a position as possible, a constant, slow stream of some weak antiseptic, such as boracic lotion, is let flow through the wound, thus preventing the accumulation of septic material, but not interfering to any great extent with the processes of repair. Drainage tubes are put into the wound, leading from the highest to counter-openings in the lowest parts, and the solution from a pail suspended over the bed is conducted to these by tubing, passes through the whole extent of the wound, and, leaving it at its most dependent part, drains into a receiver or bath placed underneath.

A special bed with a cistern for the antiseptic solution is in use for carrying out this form of treatment, the bed itself standing in a large shallow leaden trough.

By these means limbs that would otherwise inevitably have to be amputated are restored to usefulness, though the process of repair is necessarily somewhat prolonged and tedious.

## Figures and Tables

**Figure f1:**
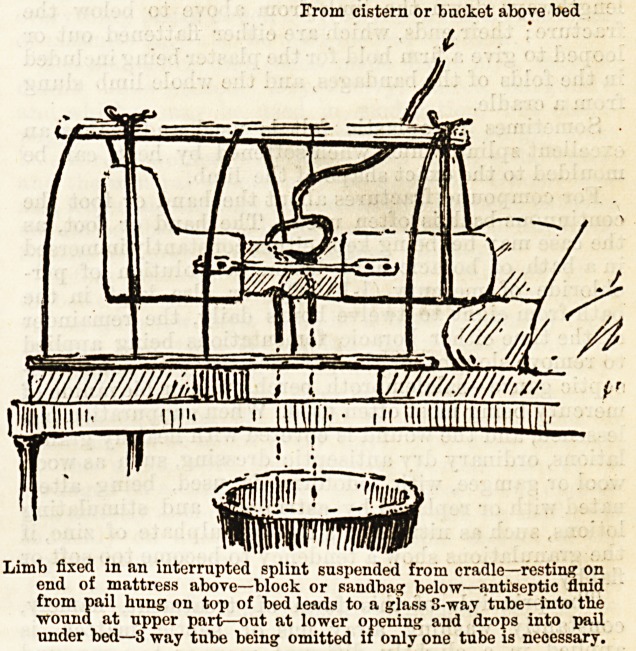


**Figure f2:**